# Repeated responders to bevacizumab combination treatment in recurrent glioblastoma: a retrospective case study

**DOI:** 10.1007/s11060-025-05162-2

**Published:** 2025-07-16

**Authors:** Maya Jeje Schuang Lü, Signe Regner Michaelsen, Alessio Locallo, Christina Schjellerup Eickhart-Dalbøge, David Scheie, Linea Cecilie Melchior, Joachim Weischenfeldt, Ulrik Lassen, Hans Skovgaard Poulsen, Thomas Urup

**Affiliations:** 1 Danish Comprehensive Cancer Center, Brain Tumor Center (DCCC-BTC), Blegdamsvej 9, Copenhagen, 2100 Denmark; 2 The Finsen Laboratory, Rigshospitalet, Ole Maaløes Vej 5, Copenhagen, 2100 Denmark; 3 Biotech Research and Innovation Center (BRIC), University of Copenhagen, Ole Maaløes Vej 5, Copenhagen, 2100 Denmark; 4 Department of Oncology, Rigshospitalet, Blegdamsvej 9, Copenhagen, 2100 Denmark; 5 The Regional Department of Clinical Microbiology, University Hospital of Region Zealand, Køge, 4600 Denmark; 6 Department of Pathology, Rigshospitalet, Blegdamsvej 9, Copenhagen, 2100 Denmark

**Keywords:** Bevacizumab, Glioblastoma, Rechallenge, Reinduction, Response

## Abstract

**Purpose:**

Bevacizumab combination treatment rechallenge in glioblastoma (GBM) patients who initially responded at recurrence has shown renewed responses in up to 60% of cases, with associated survival benefits. This study sought to characterise such repeat responders and identify predictive biomarkers.

**Methods:**

A total of 254 IDHwt GBM patients treated with bevacizumab plus chemotherapy (BevCT) were evaluated for eligibility. Five patients met the inclusion criteria for this case study by exhibiting repeated responses to BevCT and having tumour tissue available for molecular analysis. Histopathological re-assessments were performed to confirm GBM diagnoses. Angiotensinogen (AGT) promoter methylation status was analysed in all primary tumour samples, while bulk RNA sequencing and TSO500 assays were conducted on all available samples.

**Results:**

In our cohort, 40% of patients who were rechallenged with BevCT following a treatment pause exhibited a response both during the initial course and upon rechallenge. Bulk RNA sequencing revealed downregulation of HILPDA and IGF2 as potentially predictive of repeated bevacizumab response. Additionally, AGT promoter methylation analysis identified high methylation levels as another potential predictive biomarker.

**Conclusions:**

A subgroup of GBM patients responds to BevCT up to three times in the recurrent setting, and these repeated responders exhibit prolonged survival. No definitive prognostic variables or histopathological features were found in this study. Further investigation into the downregulation of HILPDA and IGF2, along with high AGT promoter methylation levels and other potential predictive biomarkers, is warranted to better understand the mechanisms underlying repeated BevCT response.

**Supplementary Information:**

The online version contains supplementary material available at 10.1007/s11060-025-05162-2.

## Introduction

Newly diagnosed glioblastoma (GBM) patients with good performance status are treated with maximal safe surgery followed by radiation therapy and temozolomide [1]. However, no standard treatment exists at disease recurrence. Multiple treatments have been tested in the recurrent setting, including re-surgery, re-irradiation, chemotherapeutic and targeted agents, but most regimens have shown limited efficacy [2]. Bevacizumab, an antibody targeting vascular endothelial growth factor A, has shown clinical activity with response rates of approximately 30% and improved progression-free survival (PFS) when combined with irinotecan or lomustine, but no improvement in overall survival (OS) when considering the total population of recurrent GBM patients [3–5]. These data demonstrate that not all patients benefit from bevacizumab combined with irinotecan or lomustine. However, a subset of recurrent GBM patients who respond to bevacizumab plus chemotherapy (BevCT) exhibit improved survival and enhanced clinical status [6–8]. This highlights the importance of characterising patients benefitting from treatment with the overall objective of identifying predictive biomarkers for efficacy. Recently, we identified low expression of the angiotensinogen (AGT) gene, regulated by methylation of its promoter region, as associated with a higher likelihood of response to BevCT [9, 10].

In patients who initially respond to BevCT, rechallenge with the therapy reportedly induces a new response in approximately 60% of cases [11–13], and even a third response in selected cases [11]. These studies reported longer survival for rechallenged patients [11, 12], indicating that re-exposure to BevCT could prolong survival in responders whose treatment was discontinued prior to tumour progression. Such findings contrast with studies testing the continuation of BevCT in patients who had previously progressed on BevCT, wherein BevCT failed to produce a second treatment response [14–16] and showed no impact on survival [16, 17].

Here, we report clinical history and pathological and genomic features of patients with progressive GBM treated with up to five individual BevCT courses resulting in clinical benefit.

## Patients and methods

### Patients

Patients in our clinical GBM database [18] who were treated with bevacizumab combined with either lomustine or irinotecan between 2007 and 2018 at Rigshospitalet for progressive IDHwt GBM were screened for eligibility. Patients were included in the study if they achieved treatment response during both the initial bevacizumab course and the rechallenge, and if GBM tissue was available for analysis. Tumour response was assessed both radiographically and clinically, in accordance with the Response Assessment in Neuro-Oncology (RANO) criteria. The control group (Table S1) used for comparison with the repeated bevacizumab responders in Kaplan–Meier plots included 38 GBM patients who had formalin-fixed paraffin-embedded (FFPE) tissue with RNA sequencing (RNA-seq) data available from either primary or relapse tumours.

### Treatment

In this retrospective cohort, first-line treatment consisted of maximal safe surgical resection followed by radiation therapy plus concomitant and adjuvant temozolomide according to the Stupp regimen [1, 18].

At time of progression, patients were evaluated by a multidisciplinary team for relapse surgery or second-line treatment. Patients with good Eastern Cooperative Oncology Group performance status (ECOG PS = 0–1) and measurable tumours on a magnetic resonance imaging (MRI) scan were offered bevacizumab combined with irinotecan or lomustine.

For patients treated with bevacizumab and irinotecan, bevacizumab was administered at 10 mg/kg every 2 weeks, alongside 125 mg/m^2^ irinotecan [19]. In the bevacizumab–lomustine group, bevacizumab was given at 10 mg/kg every 2 weeks, while lomustine was administered every 6 weeks at 90 mg/m^2^ [8].

A treatment cycle was defined as 4 weeks for bevacizumab–irinotecan and 6 weeks for bevacizumab–lomustine. A new treatment course was recorded if a new bevacizumab combination treatment was initiated after at least a 3-month pause following disease progression.

Treatment discontinuation was retrospectively attributed to documented explanations including unacceptable toxicity or patient/physician decisions. The date of treatment cessation was defined as the last recorded administration during the treatment course.

### Clinical follow-up

Patients were clinically evaluated every 2 weeks when receiving therapy, and MRI evaluation was performed after 2 cycles, being every 8 weeks in patients receiving irinotecan and every 12 weeks in patients receiving lomustine [8, 19]. Patient and tumour data were retrospectively reviewed using patient charts, pathology reports, and radiology reports. Responses were evaluated using the RANO criteria [20]. Patients were categorised according to their best response: patients with a complete response (CR) or partial response (PR) were classified as responders, whereas patients with stable disease (SD) or progressive disease (PD) were classified as non-responders. Responses (complete or partial) were defined both radiographically (≥ 50% decrease in measurably enhanced lesions) and clinically (stable or reduced corticosteroid dose; stable or improved clinical status). OS was defined both from the date of initial diagnostic surgery to the date of death, and from the start of bevacizumab combination treatment to the date of death. PFS was calculated from the date of diagnosis to the date of first progression.

### Pathology

Pathological evaluations were performed to establish GBM diagnoses in accordance with the World Health Organization (WHO) 2021 classification. In addition, DNA purified from macro-dissected FFPE tissue that was enriched for tumour content was treated with bisulfite and analysed using Infinium MethylationEPIC v1.0 BeadChip (Illumina, San Diego, CA, USA) according to the manufacturer’s instructions. Methylation profiling reports describing methylation classification, copy number variations, and O^6^-methylguanine-DNA methyltransferase (MGMT) promoter methylation status were generated using the Heidelberg Epignostix app (v12.8; app.epignostix.com), based on a previously described method [21]. AGT promoter methylation status was assessed using the generated iDAT files to analyse AGT promoter CpG site cg12469306 located in the CEBP (CCAAT/enhancer-binding protein) binding region [9].

Immunohistochemistry was used to evaluate mutations in isocitrate dehydrogenase using anti-IDH1 R132H (clone H09; 1:700; Dianova, Hamburg, Germany), and in ATRX using anti-ATRX (HPA001906; 1:150; Sigma-Aldrich, St. Louis, MO, USA). One patient was tested for H3F3A mutations using dideoxysequencing of codons 28–35.

### RNA-seq and TSO500

Archived FFPE tissue from primary tumours for cases 1, 2, 4, and 5 was used for bulk RNA-seq and TruSight Oncology (TSO) 500. For case 3, only FFPE tissue from first relapse was available. For case 5, FFPE tissue from first relapse was also available for bulk RNA-seq and TSO500. For best comparison of samples and to minimise batch effects, only FFPE tissue from the primary tumours (four samples) was evaluated in downstream differential expression analyses of bulk RNA-seq data. A control group (Table S1) consisting of 10 FFPE primary GBM tumours with available RNA-seq data was used for comparison. RNA was extracted from FFPE GBM tumour tissues from archived patient samples and sequenced using NovaSeq (Illumina). FASTQ files were processed using Nextflow [22] and the nf-core [23] pipeline (v3.8.1). STAR and Salmon were used for alignment and quantification, with Singularity (v3.6.2) as the profile option and GRCh38 as the reference genome. Downstream bulk RNA-seq analysis was performed using R software (v4.2.1; R Development Core Team, Vienna, Austria). Batch correction and differential expression analysis was performed using the R package limma (v3.52.4) [24]. Gene-set enrichment analysis was performed using the R package gprofiler2. For TSO500, all cases were evaluated relative to the most common GBM mutations [25], except for the case 5 relapse tumour sample (due to insufficient quality; Table S2).

## Results

### Clinical history of patients with repeated bevacizumab combination treatment response

A total of 254 IDHwt GBM patients treated with BevCT for progression after the Stupp regimen were screened for eligibility. As shown in the REMARK diagram (Fig. S1), 15 patients were rechallenged with BevCT after a treatment pause and six (40%) of these achieved response to both first course BevCT and BevCT rechallenge. A total of five patients with repeated BevCT response and with available tumour tissue for molecular analysis were included in the study (Table 1). Treatment covered a total of 172 BevCT cycles separated into 20 courses, including 15 courses that were rechallenges. Pauses between individual BevCT courses were for non-progression-related reasons (toxicity or patient/physician decisions) alongside MRI confirmed reduction in contrast-enhancing tumour size (response or at least SD according to the RANO criteria).

**Table 1:**
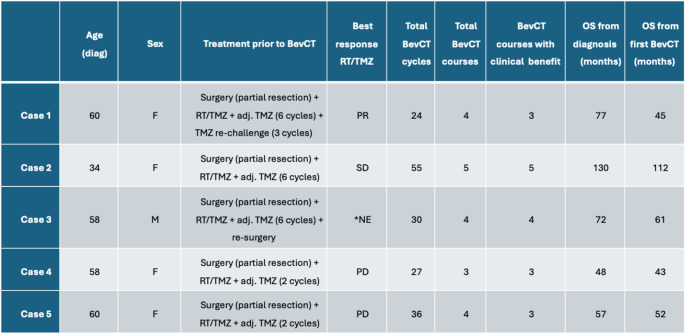
Patient characteristics of repeated BevCT responders

*NE = not evaluable according to RANO response assessment criteria

#### Case 1

**Fig. 1:**
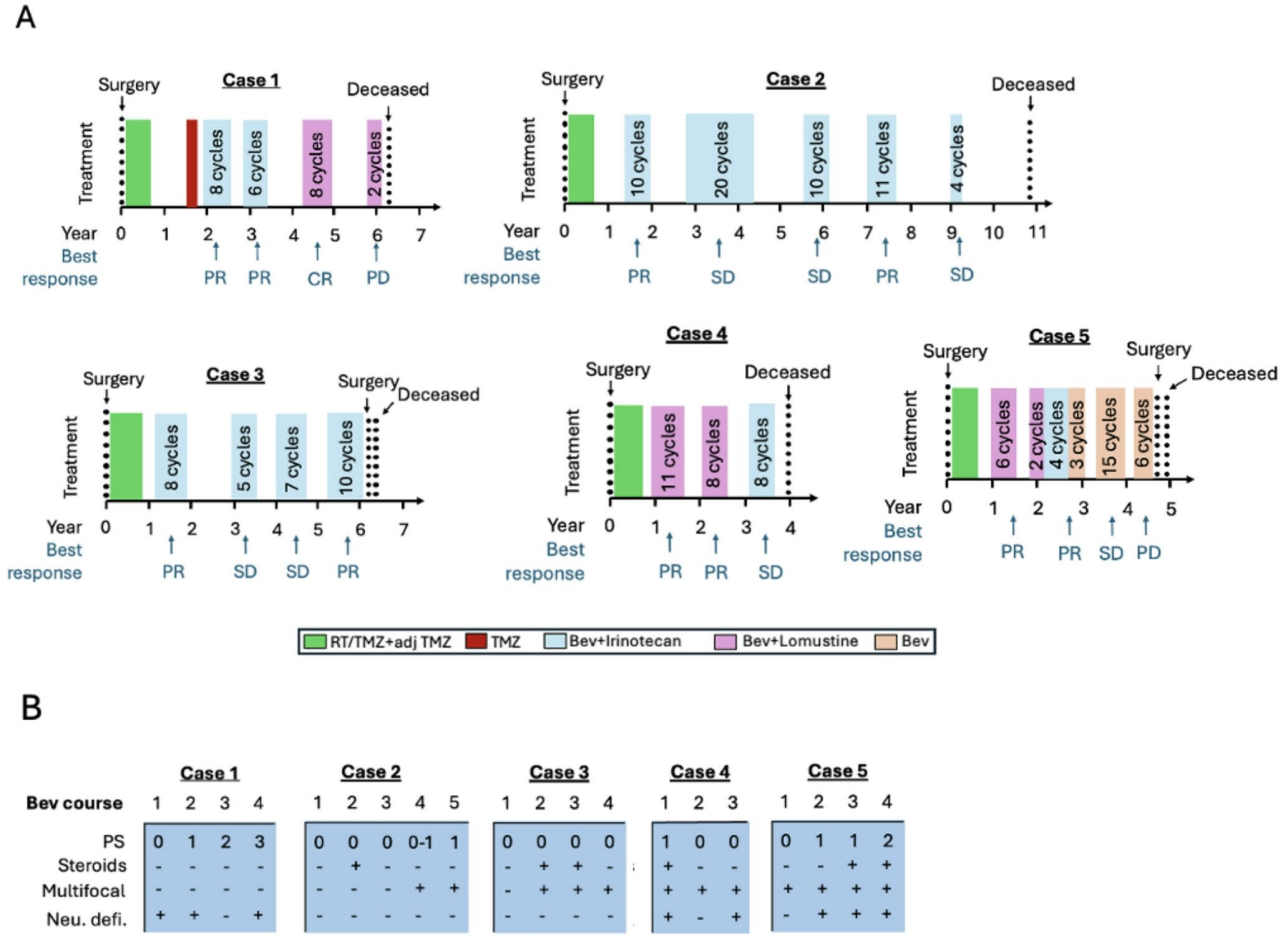
**A** Timelines showing treatments separated into cycles and courses for the repeated BevCT responders. **B** Tables representing the repeated BevCT responders, including number of courses and clinical status prior to each course: Eastern Cooperative Oncology Group performance status (PS), use of corticosteroids (Steroids), multifocal disease (Multifocal) and neurocognitive deficit (Neu. defi.). Abbreviations: Bev, bevacizumab; BevCT, bevacizumab plus chemotherapy; RT, radiotherapy; TMZ, temozolomide; CR, complete response; PR, partial response; SD, stable disease; PD, progressive disease

A 60-year-old woman with GBM, methylated MGMT (RTK2 subtype), survived 77 months after diagnosis and 45 months after initiating BevCT. Initial treatment (Fig. 1A) included partial resection followed by standard radio-chemotherapy, achieving a PR. After 16 months of SD, progression near the right lateral ventricle occurred. Temozolomide reinduction (3 cycles) failed due to further tumour progression, and bevacizumab/irinotecan was initiated.

The patient underwent 24 BevCT cycles over four courses. Prior to BevCT courses, ECOG PS declined from 0 (course 1) to 3 (course 4; Fig. 1B). Neurocognitive deficits fluctuated throughout but improved prior to course 3. No corticosteroids were used.

The first course achieved PR after two cycles, and PR remained after eight cycles, prompting a treatment pause. After 5 months, local progression led to a second course (six cycles), again with PR. After a 12-month pause a third course (bevacizumab/lomustine) achieved a durable complete response for six cycles but was paused due to clinical decline. Four months later, recurrence prompted a fourth course (bevacizumab/irinotecan), which failed due to refractory disease. The patient died shortly afterwards.

#### Case 2

A 34-year-old woman with GBM, methylated MGMT, survived 130 months from diagnosis and 112 months after starting BevCT. The patient achieved SD upon standard therapy and received 55 bevacizumab/irinotecan cycles in five courses, with 9–12 months treatment pauses.

ECOG PS was 0–1 at each course start; corticosteroids were required before course 2. No neurocognitive deficits were observed. Multifocal disease appeared before courses 4 and 5.

The first course (10 cycles) began 6 months after adjuvant therapy due to MRI-confirmed progression, achieving PR. The second course (20 cycles) achieved SD by cycle 2 and PR by cycle 6. A third course (10 cycles) achieved SD with tumour shrinkage. Course 4 was started after MRI revealed a new isolated lesion, achieving SD after two cycles and PR after 11 cycles. Progression with new tumour lesions led to a fifth course, achieving SD with lesion reduction until cycle 4, when infection and facial nerve palsy interrupted treatment. After 12 months with SD, the disease progressed with new lesions and leptomeningeal spread. The patient developed epilepsy and ventriculitis and died 5 months later.

#### Case 3

A 58-year-old man with GBM, MGMT methylated (mesenchymal subtype), survived 72 months, including 61 months after BevCT. Initial standard treatment led to PR with disappearance of contrast-enhancing lesions. At completion of adjuvant temozolomide MRI showed progression, which advanced to measurable lesions within 2 months, prompting initiation of bevacizumab/irinotecan.

The patient received 30 cycles across four courses. ECOG PS was 0 and no neurocognitive deficits were noted prior to each course. Corticosteroid use and multifocal disease were observed at later courses. The first BevCT course resulted in PR after two cycles and further tumour reduction after eight cycles, followed by a treatment pause. Eleven months later, new progressive lesions led to a second course (5 cycles), achieving SD. An infection interrupted treatment, after which tumours progressed. A third course (7 cycles) again achieved SD with tumour regression. A 17-month treatment-free interval followed. The fourth course produced durable PR, but after ten cycles progression of a new lesion prompted surgery. The patient experienced postoperative complications and died shortly thereafter.

#### Case 4

A 58-year-old woman with non-resectable, multifocal GBM (MGMT status unknown) involving the left temporal lobe and corpus callosum survived 48 months, with 43 months after BevCT. Initial treatment included standard therapy. Progression of a new lesion in the contralateral hemisphere on 3-month post-irradiation MRI prompted BevCT. The patient received 27 BevCT cycles over three courses. Prior to course 1, ECOG PS was 1 and the patient used corticosteroids. Before subsequent courses, PS had improved to 0 and corticosteroids were no longer needed. The first course led to clinical improvement and PR after two cycles, with further tumour reduction after nine cycles. Treatment was paused after 11 cycles but a new measurable lesion appeared 6 months later. A second course (bevacizumab/lomustine) achieved durable PR for eight cycles but treatment was interrupted due to grade 3 hepatic enzyme elevation attributed to lomustine. Following a 5-month pause, MRI showed progression, and a third course (bevacizumab/irinotecan) was initiated, achieving SD over eight cycles. The patient clinically deteriorated 2 months after the final pause and died shortly thereafter.

#### Case 5

A 60-year-old woman with GBM, MGMT methylated (mesenchymal subtype), located in the left occipital and right parietal lobe survived 57 months, with 52 months after BevCT. Standard therapy was followed by early progression (focal seizures and two new lesions), leading to BevCT.

The patient received 36 cycles over four treatment courses. Multifocal disease was present from the outset. ECOG PS was 0–1 at courses 1 and 2, declining to 2 at course 4. Corticosteroids were used prior to courses 3 and 4. The first course with bevacizumab/lomustine achieved durable PR for six cycles until paused due to grade 3 thrombocytopenia. After a 5-month treatment pause, progression prompted reinduction with lomustine (discontinued due to thrombocytopenia) and a switch to irinotecan. A second durable PR was achieved during 6 cycles of BevCT and 3 cycles of bevacizumab monotherapy. Following a 3-month pause, progression led to 15 additional bevacizumab monotherapy cycles, with SD as the best response. A final rechallenge after another 5-month break resulted in progression after six cycles. Re-surgery confirmed recurrent GBM (RTK2 subtype). The patient died 3 months later.

### Pathological examinations to confirm GBM diagnoses

For all five cases, initial GBM diagnoses were based on histological features such as pleomorphic cells, necrosis, vascular proliferation and mitotic indices. Molecular analyses supported the GBM diagnoses at the time (2008–2013), but we reviewed the material with up-to-date pathological methods to confirm diagnoses in relevant cases (Table 2). Copy number plots derived from the methylation analysis was used to evaluate 1p/19q codeletion status and characteristic copy number alterations (-+7/-10, EGFR amplification). Common GBM mutations, including TERT promoter mutations, were assessed via TSO500 (Table S2). All included cases met the diagnostic criteria for IDHwt GBM according to the 2021 WHO classification. AGT promoter methylation status, which we previously identified as a predictive biomarker for bevacizumab response [9], was analysed in the four cases with primary diagnostic tumour tissues. In these cases, the AGT promoter was more methylated (median: 26%; range: 16–49%) than our previously identified cut-off point of 12% [9], indicating these patients may be more likely to respond to BevCT.

**Table 2:**
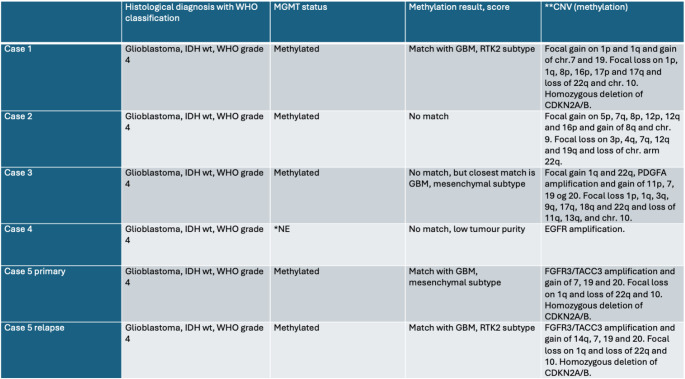
Pathology data on repeated BevCT responders

*NE = not evaluable due to low tumour purity

### Bulk RNA-seq and TSO500

**Fig. 2:**
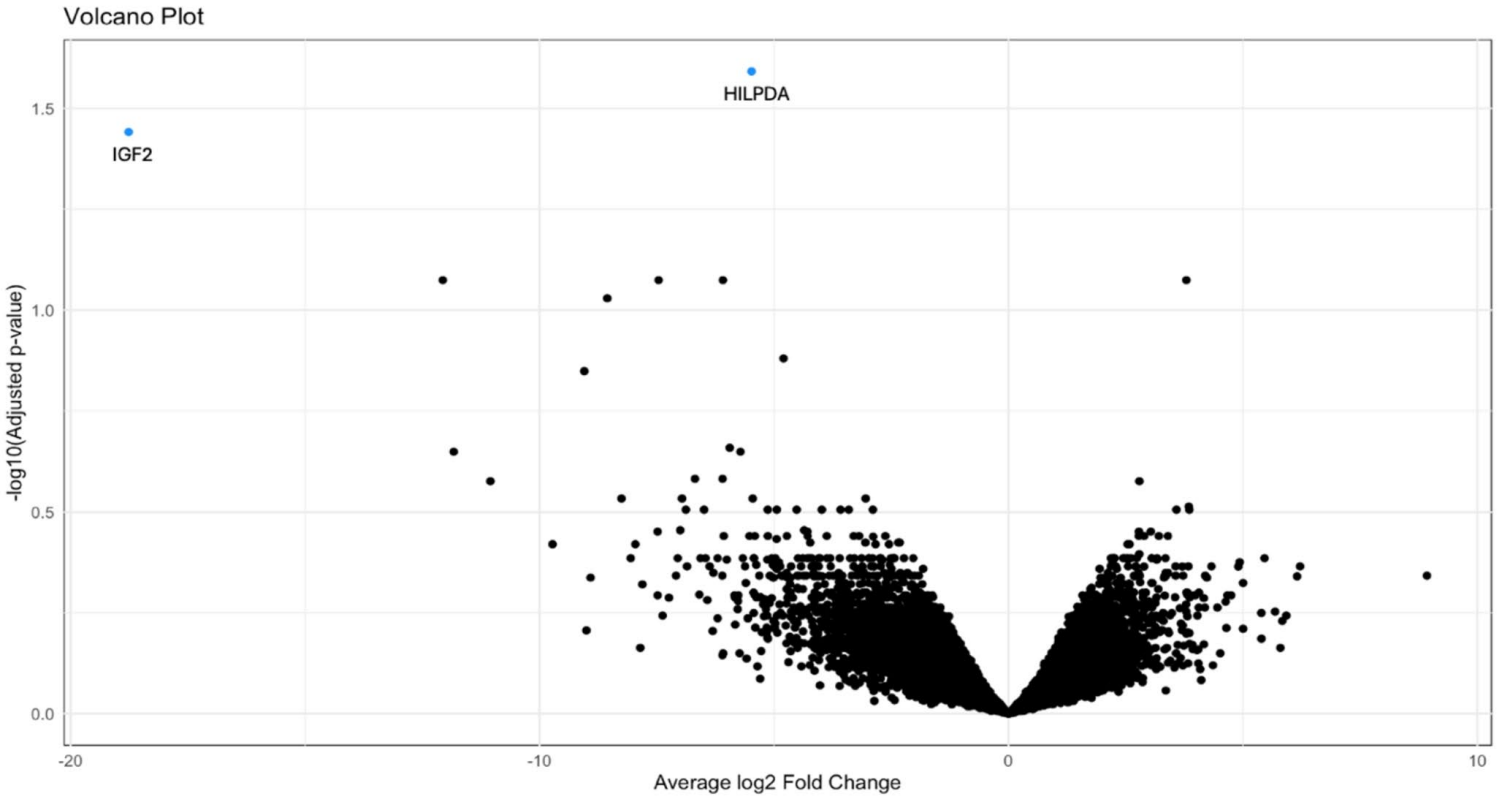
Volcano plot showing two significantly downregulated genes HILPDA and IGF2 (dodgerblue) in primary patient samples from four repeated BevCT responders compared to 10 control samples. X-axis: Average log2 Fold Change. Y-axis:–log10 transformed adjusted *p*-values

Differential expression analysis on bulk RNA-seq data showed significant downregulation of two genes: HILPDA (logFC, − 5.47; adjusted *p*-value, 0.026) and IGF2 (logFC, − 18.76; adjusted *p*-value, 0.036) in the four repeated BevCT responders compared to the 10 controls. The genes are blue in the volcano plot (Fig. 2). TSO500 data showed no specific mutation pattern in the most common GBM-mutated genes (e.g., TERT, PTEN, EGFR and TP53; Table S2).

## Discussion

We describe five recurrent GBM cases showing clinical benefit from BevCT rechallenge up to five times, after treatment was stopped for reasons other than disease progression. This surpasses the one to two rounds of successful BevCT rechallenges previously reported for GBM [11]– [12]. Together with other reports of exceptionally high response rates to BevCT rechallenge in previously responding patients following non-progression-related treatment pauses [11]– [12], our data suggest a rare subgroup of GBM tumours may be highly sensitive to BevCT.

**Fig. 3:**
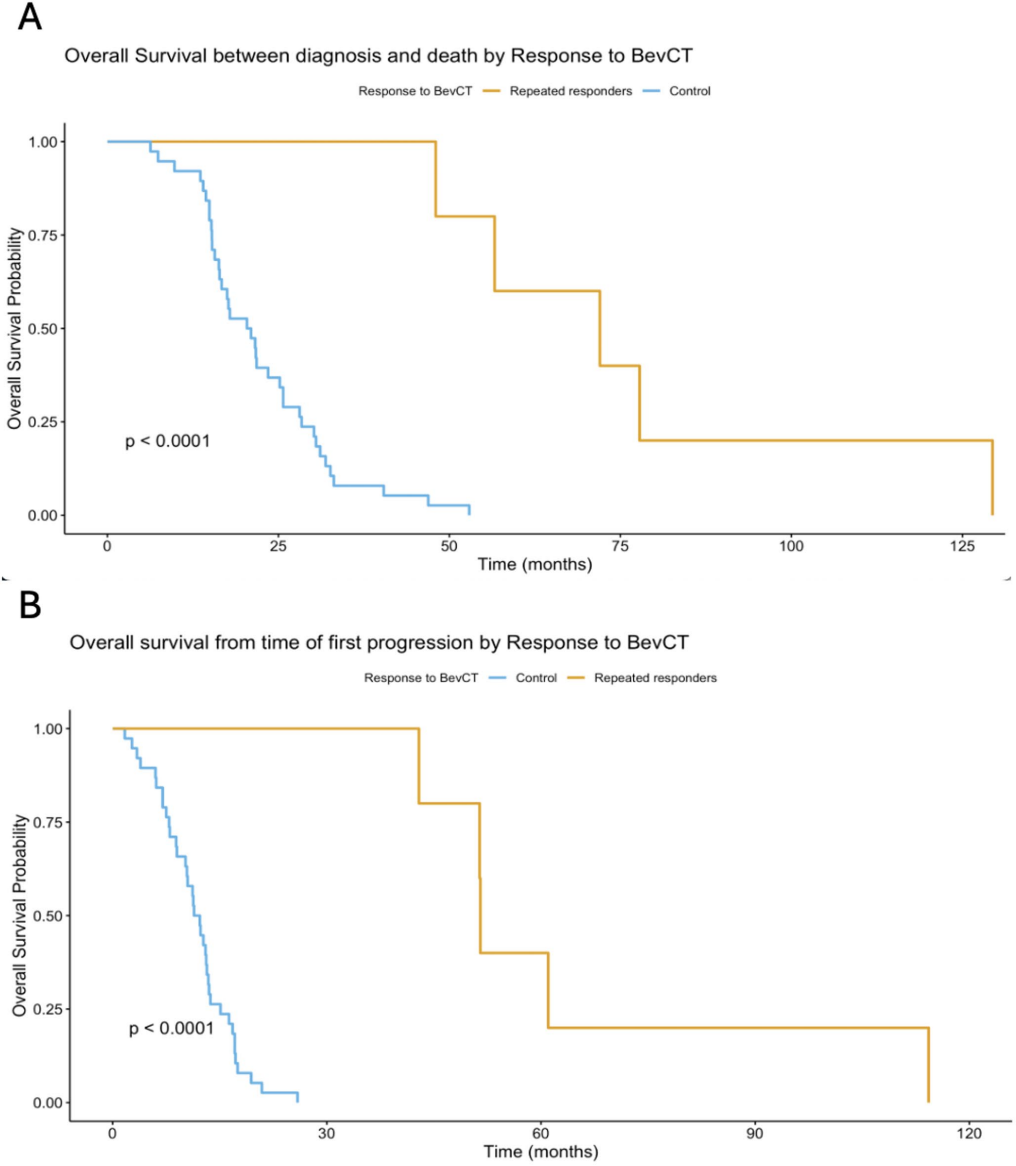
**A** Kaplan–Meier curves showing overall survival between date of diagnosis and death in BevCT repeated responders (orange) and controls (light blue). **B** Kaplan–Meier curves showing overall survival between relapse and death in BevCT repeated responders (orange) and controls (light blue)

All five patients exhibited OS (from diagnosis) far beyond the median of 15 months (Fig. 3A) and OS from start of relapse treatment far beyond the median of 8–9 months (Fig. 3B) normally observed for GBM patients [4]– [5, 19]. Negative phase III clinical trials showing no survival benefit of BevCT compared to chemotherapy alone [4, 26] have prompted speculation that MRI-apparent bevacizumab responses do not reflect true anti-tumour effects. Instead, these may represent pseudo-responses caused by bevacizumab-induced reductions in vascular permeability, leading to decreased contrast enhancement on MRI [27]. Furthermore, bevacizumab may trigger rebound progression characterised by a fast-growing phenotype [28]. Multiple mechanisms of acquired bevacizumab resistance have been proposed [29], including a phenotypic shift from vascular growth to an invasive growth pattern, potentially contributing to more rapid progression [30]– [31]. However, our findings, aligned with previous studies [11]– [12], suggest that these explanations may not be applicable for all patients. The observed combination of repeated bevacizumab responses and prolonged survival in some cases indicates that a subset of GBM patients may exhibit true anti-tumour effects from bevacizumab combination treatment.

An important consideration is whether the repeated responders in our study have accurate GBM diagnoses. Therefore, we performed comprehensive histopathological and molecular re-evaluation of all cases, confirming their classification as IDHwt GBM according to the 2021 WHO criteria.

Another consideration is whether the favourable outcomes in our study cohort could reflect the underlying biology of long-term survivors (LTSs), a rare subset of GBM patients who survive > 5 years after diagnosis. However, due to the absence of consistent clinical or molecular biomarkers defining LTSs, this potential confounder cannot be definitively excluded. Nevertheless, in contrast to LTSs, whose extended survival is associated with prolonged remission following standard therapy [32], our cohort of BevCT responders showed similar PFS to the control group (Fig. S2). Moreover, previous studies have not found an association between response to first-line treatment and bevacizumab efficacy [19]. Collectively, these findings suggest that the favourable outcomes observed in our cohort are not solely attributable to inherently favourable tumour biology. Instead, they support the hypothesis that a subset of tumours may possess intrinsic susceptibility to bevacizumab-based treatment.

Among the traits characteristic of this small patient cohort, primary tumour tissues exhibited methylation levels of the AGT promoter that ranged from 16 to 49%, which is above our previously identified and validated cut point of 12% associated with response to BevCT [9]. This supports our previous hypothesis that AGT gene silencing by promoter methylation may be a predictive biomarker for response to BevCT [9], currently being tested in a randomised phase III trial (EudraCT No. 2020-003545-11). Bulk RNA-seq revealed significant downregulation of HILPDA and IGF2 at the gene level, but not at the pathway level. HILPDA has been linked to a hypoxic phenotype in gliomas [33]– [34] and a less hypoxic environment may be more likely to undergo bevacizumab-induced vascular normalisation than more hypoxic and necrotic tumours [35]– [36]. Whether the HILPDA and IGF2 genes are predictive biomarkers of BevCT response must be tested in a larger prospective cohort.

In our study, analysis of gene alterations and methylation-based subclasses revealed no consistent traits among BevCT responders. Regarding, previously reported independent prognostic variables for BevCT treated GBM (ECOG PS, tumour multifocality, corticosteroid use and neurocognitive deficit) [19, 37]– [38], our cases overall showed good prognostic status before each effective BevCT course. At least four of the five patients had MGMT promoter methylated GBM tumours as well. These factors may have contributed to the favourable outcomes but do not fully explain the sustained benefit observed after multiple BevCT rechallenges.

Taken together, these findings suggest the presence of a subset of GBM patients with a distinct biological profile that confers heightened sensitivity to bevacizumab-based therapy. However, given the small cohort size, the retrospective study design, non-matched control group, and the potential confounding effect of LTS biology, validation studies are needed to draw solid conclusions.

## Conclusions

GBM patients can exhibit repeated responses to BevCT in the recurrent setting after a treatment pause. These repeated responders are notable for exhibiting extended survival, suggesting that a small subgroup of GBM patients may be sensitive to BevCT. In this study, no specific prognostic variables or histopathological features distinguished the responders from other GBM patients. However, HILPDA and IGF2 were downregulated compared with the controls. Due to the small sample size, further studies are necessary to determine whether downregulated gene expression of HILPDA and IGF2 and hypermethylation of the AGT promoter could be predictive biomarkers of BevCT response.

## Electronic supplementary material

Below is the link to the electronic supplementary material.

Supplementary Material 1

## Data Availability

No datasets were generated or analysed during the current study.
