# Supplementary material for: Repeated responders to bevacizumab combination treatment in recurrent glioblastoma: a retrospective case study
Source: J Neurooncol. 2025 Jul 16;175(2):869–78. doi: 10.1007/s11060-025-05162-2 (PMC12420756; doi:10.1007/s11060-025-05162-2)
Supplement: Supplementary file 1 — Supplementary Material 1 [file 11060_2025_5162_MOESM1_ESM.docx]

**Supplementary material**

**
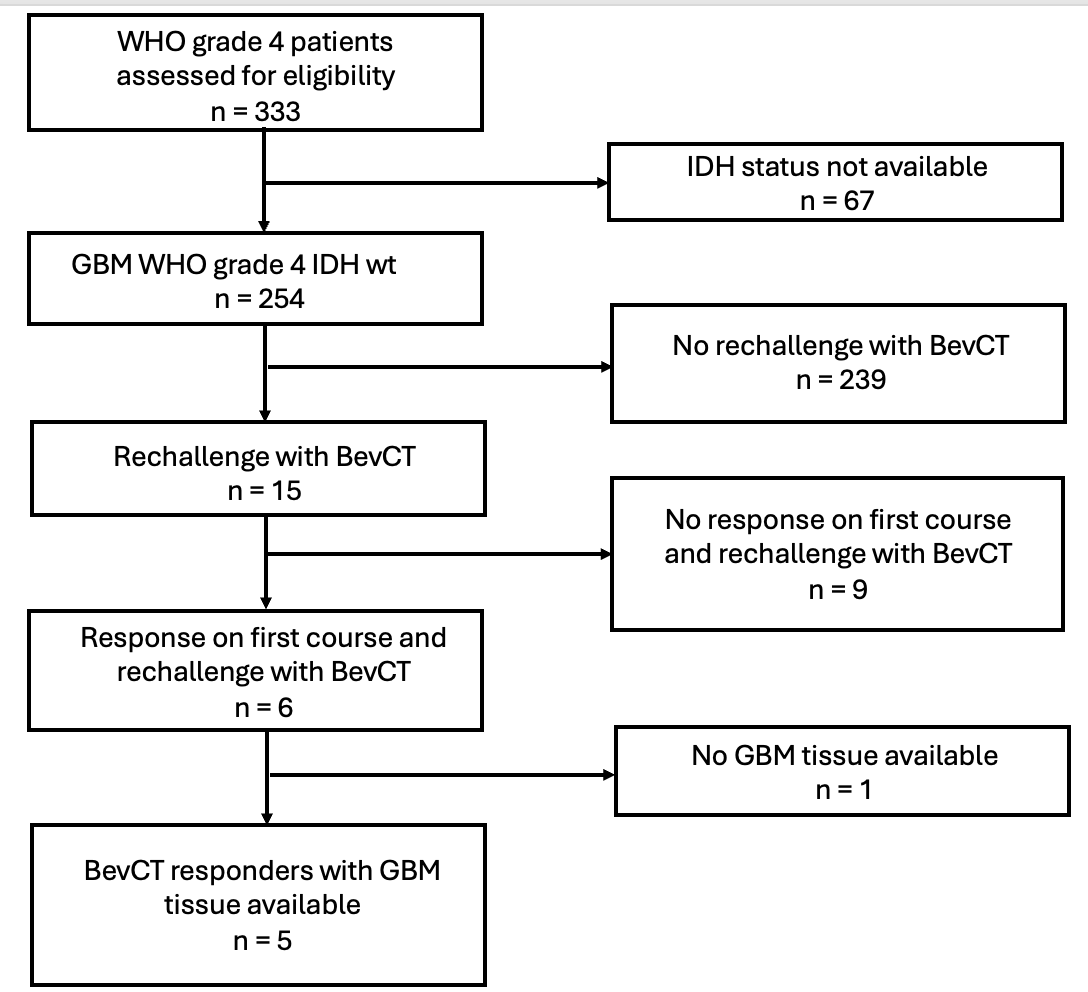
**

**Figure S1:** REMARK diagram. Abbreviations: BevCT, bevacizumab plus chemotherapy; IDHwt, isocitrate dehydrogenase wild-type; WHO, World Health Organization; GBM, glioblastoma.


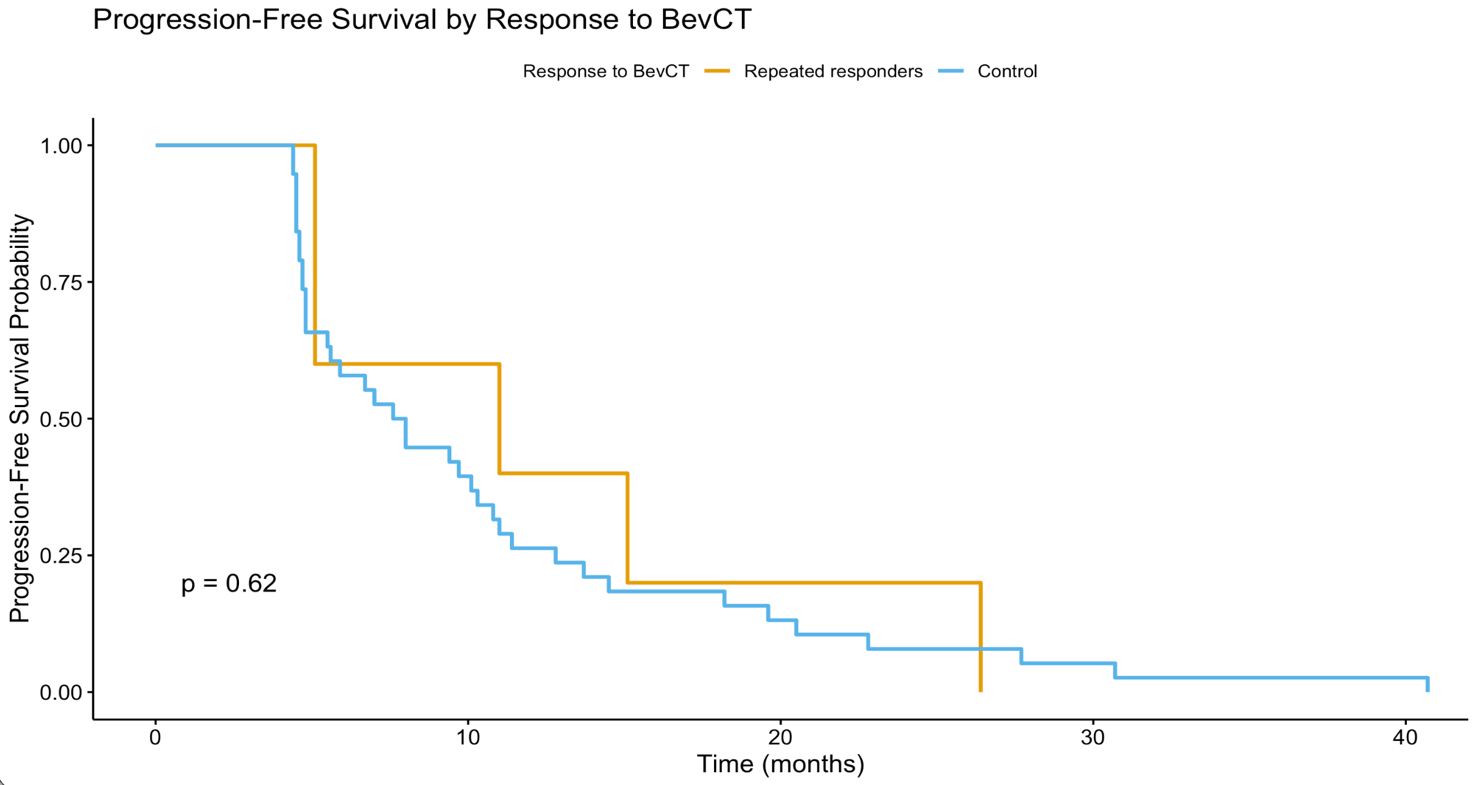


**Figure S2:** Kaplan–Meier curves showing progression-free survival between diagnosis and first relapse in BevCT repeated responders (orange) and controls (light blue).

**Table S1:** Patient characteristics of the Responder and The FFPE control cohort, including the Primary FFPE control cohort used for RNA-seq analysis

| Patient characteristics prior to first-line treatment | | | |
| --- | --- | --- | --- |
|  | Repeated  responder cohort  (*n* = 5) | FFPE control  cohort  (*n* = 38) | Primary FFPE  control cohort  (*n* = 10) |
| **Median age, years (range)** | 58 (34–60) | 59 (33–76) | 57 (33–70) |
| **Sex, *n* (%)** |  |  |  |
| Male | 1 (20%) | 24 (63%) | 8 (80%) |
| Female | 4 (80%) | 14 (37%) | 2 (20%) |
| **ECOG performance status, *n* (%)** |  |  |  |
| 0 | 4 (100%) | 24 (67%) | 6 (60%) |
| 1 | 0 (0%) | 10 (28%) | 4 (40%) |
| 2 | 0 (0%) | 2 (5%) | 0 (0%) |
| Missing | 1 (20%) | 2 (5%) | 0 (0%) |
| **Multifocal disease, *n* (%)** |  |  |  |
| Yes | 2(40%) | 1 (3%) | 0 (0%) |
| No | 3(60%) | 37 (97%) | 10 (100%) |
| **Tumour resection, *n* (%)** |  |  |  |
| Biopsy | 0 (0%) | 4 (11%) | 3 (30%) |
| Surgical resection | 5 (100%) | 34 (89%) | 7 (70%) |
| **MGMT status, *n* (%)** |  |  |  |
| Methylated | 4 (100%) | 13 (34%) | 2 (22%) |
| Unmethylated | 0 (0%) | 25 (66%) | 9 (78%) |
| Missing | 1 (20%) | 0 (0%) | 1 (10%) |
| **Corticosteroid use, *n* (%)** |  |  |  |
| Yes | 4 (80%) | 13 (34%) | 2 (20%) |
| No | 1 (20%) | 25 (66%) | 8 (80%) |
| **Resection at time of recurrence, *n* (%)** |  |  |  |
| Yes | 2 (40%) | 32 (84%) | 5 (50%) |
| No | 3(60%) | 6 (16%) | 5 (50%) |
| **Second-line treatment, *n* (%)** |  |  |  |
| Yes | 5 (100%) | 34 (89%) | 9 (90%) |
| No | 0 (0%) | 4 (11%) | 1 (10%) |
| **Bevacizumab containing relapse treatment, *n* (%)** |  |  |  |
| Yes | 5 (100%) | 25 (66%) | 8 (80%) |
| No | 0 (0%) | 13 (34%) | 2 (20%) |

Abbreviations: FFPE, formalin-fixed paraffin-embedded; ECOG, Eastern Cooperative Oncology Group; MGMT, O^6^-methylguanine-DNA methyltransferase.

**Table S2:** Table of common glioblastoma mutations from TSO500 panel of all five repeated BevCT responders.


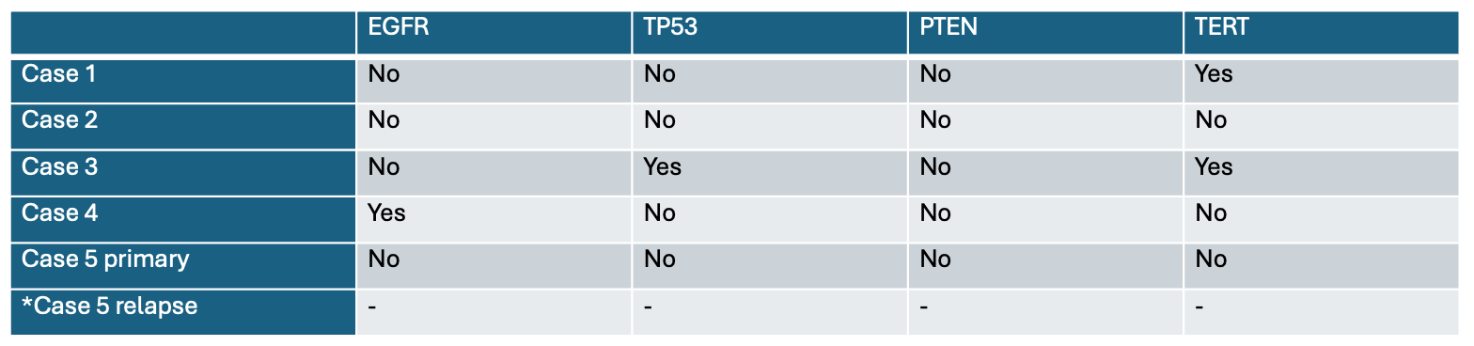


*TSO500 of insufficient quality to evaluate mutations
